# Role of Cav2.3 (R-type) Calcium Channel in Pain and Analgesia: A Scoping Review

**DOI:** 10.2174/1570159X21666230811102700

**Published:** 2023-08-15

**Authors:** Marcella de Amorim Ferreira, Juliano Ferreira

**Affiliations:** 1 Graduate Program of Pharmacology, Universidade Federal de Santa Catarina, Florianopolis, SC, Brazil

**Keywords:** VGCC, Cav2.3, calcium channel r-type, *CACNA1E*, pain, analgesia

## Abstract

**Background:**

Voltage-gated calcium channels (VGCCs) play an important role in pain development and maintenance. As Cav2.2 and Cav3.2 channels have been identified as potential drug targets for analgesics, the participation of Cav2.3 (that gives rise to R-type calcium currents) in pain and analgesia remains incompletely understood.

**Objective:**

Identify the participation of Cav2.3 in pain and analgesia.

**Methods:**

To map research in this area as well as to identify any existing gaps in knowledge on the potential role of Cav2.3 in pain signalling, we conducted this scoping review. We searched PubMed and SCOPUS databases, and 40 articles were included in this study. Besides, we organized the studies into 5 types of categories within the broader context of the role of Cav2.3 in pain and analgesia.

**Results:**

Some studies revealed the expression of Cav2.3 in pain pathways, especially in nociceptive neurons at the sensory ganglia. Other studies demonstrated that Cav2.3-mediated currents could be inhibited by analgesic/antinociceptive drugs either indirectly or directly. Some articles indicated that Cav2.3 modulates nociceptive transmission, especially at the pre-synaptic level at spinal sites. There are studies using different rodent pain models and approaches to reduce Cav2.3 activity or expression and mostly demonstrated a pro-nociceptive role of Cav2.3, despite some contradictory findings and deficiencies in the description of study design quality. There are three studies that reported the association of single-nucleotide polymorphisms in the Cav2.3 gene (CACNA1E) with postoperative pain and opioid consumption as well as with the prevalence of migraine in patients.

**Conclusion:**

Cav2.3 is a target for some analgesic drugs and has a pro-nociceptive role in pain.

## 1 INTRODUCTION

Noxious stimuli are detected in the periphery by primary afferent nerve fibers, called nociceptors, and then this stimulus is transmitted to CNS. There are two main classes of nociceptors: Aδ myelinated primary afferent fibers and C unmyelinated primary afferent fibers. The cell bodies of nociceptors are located in DRG. Primary afferent nerve fibers project to the dorsal horn of the spinal cord and stimulate the depolarization and release of neurotransmitters, such as glutamate, substance P and CGRP. These projection neurons are at the origin of multiple ascending pathways, including the spinothalamic and spinoreticulothalamic tracts, which carry pain messages to the thalamus and brainstem, respectively. In situations of persistent nociception or neuroplasticity after lesion, there are changes in transmission and modulation of pain. In chronic pain conditions, the activity of nociceptors is increased, nociceptive transmission is modified, there are changes in action potential firing, increase in the activation of voltage-gated channels (sodium and calcium), increased release of neurotransmitters, and aberrant activation of kinases and glial activities [1-3].

Chronic pain can be a debilitating condition that affects approximately 20 percent of adults and is a major cause of demand for health services. In 2013, pain conditions were the third cause of health spending, and in 2016, Americans spent 380 billion dollars on pain treatments [4]. Between 2007 to 2017 in USA, two of the ten most debilitating health problems were related to pain conditions (Global Burden of Disease, 2017). The International Association for the Study of Pain (IASP) defines pain as “An unpleasant sensory and emotional experience associated with, or resembling that associated with, actual or potential tissue damage” [5]. Thus, pain is not just the neural process of encoding noxious stimuli (nociception), but is always a personal experience that is influenced to varying degrees by biological, psychological, and social factors [6].

Pain can range widely in intensity, quality, and duration and arises from diverse pathophysiological mechanisms. The IASP classifies pain as nociceptive pain (pain arising from activation of nociceptors, including inflammatory pain), nociplastic pain (pain arising from altered nociception despite no clear evidence of actual or threatened tissue damage or evidence for disease or lesion of the somatosensory system) and neuropathic pain (pain caused by a lesion or disease of the somatosensory nervous system) [5]. There are also mixed forms of pain, with patients demonstrating nociceptive and neuropathic pain characteristics, such as cancer pain and post-operative pain [7]. Some patients may develop allodynia (pain in response to a non-nociceptive stimulus) and hyperalgesia (increased pain sensitivity to nociceptive stimulus), in addition to spontaneous pain [8].

Pain may also be classified according to anatomical localization, as somatic pain (superficial, well localized, reaching subcutaneous tissues, muscles and joints) or visceral pain (deep, diffuse and reach viscera) [9]. Based on its duration, pain can be described as acute and chronic. Acute pain starts immediately after injury, has limited duration, and has a temporal and causal relationship with the lesion or disease. In some individuals, there can be a transition from acute to chronic pain that is defined by persisting for at least 3 months [3], with underlying molecular mechanisms that are still not fully understood.

Analgesia can be defined as the absence of pain in response to stimulation which would normally be painful and may be achieved by non-pharmacological and pharmacological (analgesic drugs) management. Different types of pain may respond better to different analgesic drugs. For example, neuropathic pain may respond best to tricyclic antidepressants, serotonin and norepinephrine reuptake inhibitors (SNRIs) or anticonvulsants than to opioids [10]. In addition, the use of opioids is limited by side effects, including nausea, constipation, and respiratory depression, as well as the potential for analgesic tolerance and dependence with chronic use [11]. The use of nonsteroidal anti-inflammatory agents (NSAIDs) is indicated for acute inflammatory pain. However, NSAIDs do not work well for neuropathic pain treatment, and careful dosage compliance is essential to avoid gastric ulcer formation and harmful effects on renal function [12]. Accordingly, novel, effective, safe treatment approaches to pain treatment are urgently needed.

Various animal models of pain have been developed that recapitulate the diverse symptoms of different pain pathologies [13]. Such animal models have aided in our understanding of the pathophysiological mechanisms responsible for the generation of pain and allowed us to identify and validate effective and safe analgesic drug candidates [14]. However, animal pain models have limitations, including study design quality, reproducibility, predictive validity as well as detection of nociception instead pain [15].

Voltage-gated calcium channels (VGCCs) are important mediators of calcium influx into electrically excitable cells such as neurons. These channels are expressed in most plasma membrane compartments, and they are required for a plethora of cellular functions, including regulation of neurotransmitter release, hormone secretion, genic transcription, and others [16]. VGCCs are activated by membrane depolarization, and are classified according to their electrophysiological and pharmacological characteristics into high-voltage-activated channels (HVAs) that require large membrane depolarizations for opening and low-voltage activated (LVA) that open at more negative voltages. The HVA channels consist of L-type (long-lasting currents), P/Q-type (first described in Purkinje cells), N-type (found mostly in neuronal tissues), and R-type channels (which produce pharmacologically resistant currents). LVA channels encompass the T-type (transitory currents) channels [17, 18]. HVA channels are comprised of a pore-forming (α1) subunit and auxiliary subunits (α2-δ, β, and γ), whereas LVA channels only require the α1 subunit to be functional [17]. The mammalian genome encodes 10 different α1 subunits, which are named and subdivided into families based on their homology: family 1 (Cav1.1-1.4) generates L-type calcium currents, members of the Cav2 family mediate P/Q-type (Cav2.1), N-type (Cav2.2), and R-type (Cav2.3) currents, and family 3 (Cav3.1-3.3) generates T-type currents (Dolphin 2016). The members of the Cav2 family are found mostly in neuronal tissue, especially in pain pathways, such as the small diameter sensory neurons and the dorsal horn of the spinal cord, suggesting that they play an important role in nociception [17].

There are several examples of clinically active analgesics that target N-type calcium channel activity. Gabapentinoids (*e.g*., gabapentin and pregabalin) are analgesic drugs that target the auxiliary α2δ subunits of VGCCs thereby decreasing N-type calcium current amplitude as a result of altered channel trafficking [19]. There are four subtypes of α2δ’ subunits (α2δ-1 to 4), and gabapentinoids target both α2δ-1 and α2δ-2 subtypes which are known to associate with HVA channels [17]. Ziconotide (Prialt^®^) is a synthetic version of ω-conotoxin MVIIA that provides selective and potent Cav2.2 inhibition [20]. Intrathecal ziconotide was found to produce strong antinociceptive effects in a broad array of animal models and, after three positive randomized control trials, is now approved by FDA for the treatment of severe chronic pain [21, 22]. Finally, the activation of µ-opioid receptors is known to lead to inhibition of Cav2.2 channel activity *via* direct binding of G protein βγ subunits, which along with activation of G protein-coupled inward rectifier potassium channels, triggers analgesia [23]. Collectively, these examples highlight the clinical utility of N-type calcium channels as targets for analgesics. In addition to Cav2.2, there is now a large body of evidence for a key role of Cav3.2 T-type calcium channels in pain signalling, and numerous studies have highlighted these channels as a potential target for analgesics in preclinical models (reviewed recently by Snutch and Zamponi, 2018, Harding and Zamponi, 2022).

Unlike Cav2.2 and Cav3.2, the role of Cav2.3 in painful signalling is less clear. Cav2.3 channels are distributed in tissues associated with pain detection, transmission, and modulation, including sensory neurons, DRG, trigeminal ganglia and periaqueductal grey [24-26]. In the CNS, Cav2.3 participates in synaptic plasticity and neurotransmitter release [27]. Despite the evidence of the expression of Cav2.3 in nociceptive pathways, the literature that relates Cav2.3 and pain and analgesia remains sparse. Therefore, we conducted a scoping review to map research in this area, as well as to identify any existing gaps in knowledge.

## 2 MATERIALS AND METHODS

We conducted our literature review using the PRISMA Extension for Scoping Review (PRISMA-ScR) [28]. Inclusion criteria were all years of publication, written in English, and work conducted in human individuals, laboratory animals or cell culture. Studies with any pain outcome or using any analgesic/antinociceptive drugs were included, and other outcomes were excluded [29] (See below).

To identify potentially relevant documents, we searched PubMed and SCOPUS databases with the aid of librarians. We performed searches up to May 2022. The final search results were exported into Mendeley, and the search terms used are listed in Table **1**.

Two reviewers (M.A.F and J.F.) working sequentially evaluated the titles, abstracts and then the full text of all publications identified by our searches for relevance. If needed, we resolved disagreements on study selection and data extraction by consensus and discussion with other reviewers.

1. Original papers, clinical and non-clinical, are included.
2. All populations are included.
3. Studies using Cav2.3 in any pain outcome are included.
4. Review articles are excluded (as they contain no primary data).
5. Studies not written in the English language are excluded.
6. Studies on other calcium channels that do not address Cav2.3 are excluded.
7. Studies on Cav2.3 that are not relevant to pain behavior or pain pathways are excluded.

**Fig. (1):**
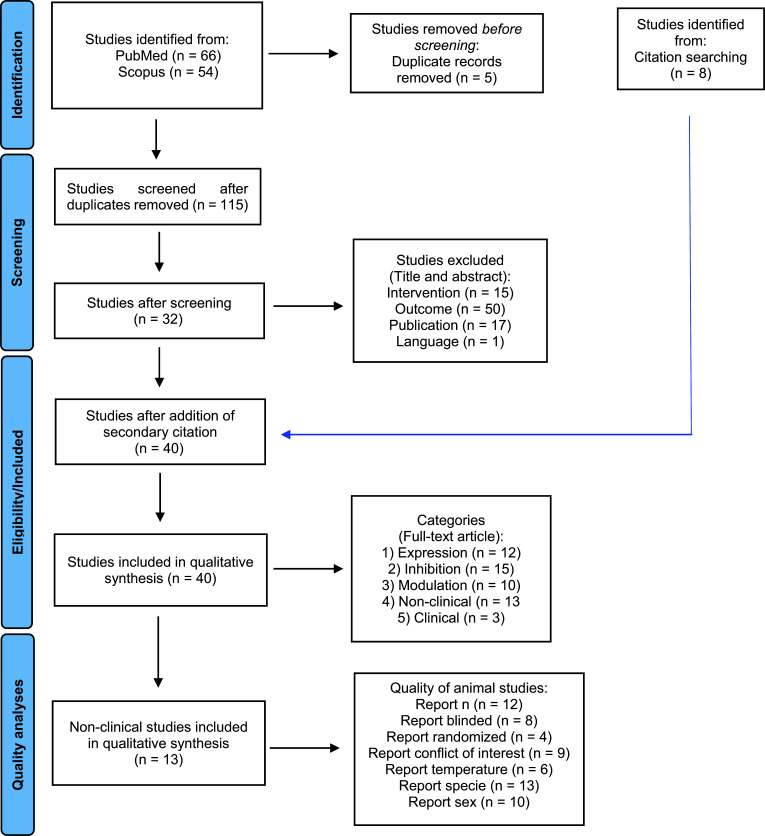
Prisma flow diagram.

We identified 66 studies in PubMed and 54 in SCOPUS, with 5 studies being duplicated in both data sets (Fig. **1**). For these 115 studies, we applied exclusion criteria based on the title and the abstract, leading to the elimination of 15 studies because they dealt with channels other than Cav2.3. In addition, 50 studies were excluded because the outcome was not associated with any pain behavior or pathways. We excluded 17 further studies as they did not constitute original research. Further, we excluded one study written in Chinese. Thus, 32 studies remained for deeper analysis. After carefully reading the whole text, 8 additional studies were identified by analysis of cited literature. Thus, we identified 40 studies that are eligible for this scoping review (Fig. **1**).

To enhance the organization of our hits, we grouped the studies as follows; these 40 articles were organized into 5 types of categories within the broader context of the role of Cav2.3 in pain and analgesia: 1) Expression of Cav2.3 in pain pathways and its alteration by painful conditions; 2) Direct or indirect inhibition of R-type currents by analgesic/antinociceptive drugs; 3) Cav2.3 modulation of nociceptive transmission; 4) Cav2.3 modulators on non-clinical (animal) models of pain and 5) Cav2.3 polymorphism association with clinical pain conditions. Additional groupings were done according to criteria such as type of population, pain outcomes, Cav2.3 focus, pain models used, intervention and some quality criteria (n, randomized, blinded, conflict of interest, sex, temperature, species) [30].

## 3 RESULTS AND DISCUSSION

### 3.1 Expression of Cav2.3 in Pain Pathways and its Alteration by Painful Conditions

The expression of Cav2.3 in pain pathways was predominantly reported in sensory ganglia (especially DRG of rodents, but also in humans), both at the mRNA and protein levels, typically in neuronal cell membranes (and occasionally in satellite cells) (Table **2**). At the sensory ganglia level, Cav2.3 is co-expressed with different somatosensory neuron markers, especially but not exclusively, in the cell bodies of nonpeptidergic (IB4+) and peptidergic (IB4- and SP or TrkA+) unmyelinated C-nociceptive neurons. Moreover, Cav2.3 was co-expressed with several receptors and channels that are targets for analgesic drugs, including TRPV1, GABA_B_R, Cav2.2 and NTSR2. Both down and up-regulation of Cav2.3 expression were reported in rodent models of cancer-related pain or inflammatory visceral hypersensitivity, respectively. Cav2.3 expression was also detected at the superficial laminae of the dorsal horn of the spinal cord in presynaptic membrane fractions, an anatomical region known to be critical for the first stage processing of noxious and thermal stimuli and that contains the synaptic terminals of nociceptive neurons [31]. A strong upregulation of Cav2.3 mRNA in the spinal cord after peripheral nerve injury in rats has been reported, an effect that was curiously not observed after injury in mice at the protein level (Table **2**). Cav2.3 was also detected in supraspinal structures that are very important in the processing of nociceptive information, including the thalamus, hypothalamus, amygdala and periaqueductal gray where sensory-discriminative, endocrine and affective dimensions of pain are processed [32-35]. We were not able to find any alteration of Cav2.3 expression in supraspinal structures in painful conditions (Table **2**) [36-46]. Finally, the expression of Cav2.3 protein in intrinsic primary afferent neurons of guinea pig intestine was reported. These are the first neurons of the intrinsic reflexes and may be involved in the analgesic effect of gabapentinoids in inflammatory bowel diseases (Table **2**).

### 3.2 Direct or Indirect Inhibition of R-type Currents by Analgesic/Antinociceptive Drugs

**Fig. (2):**
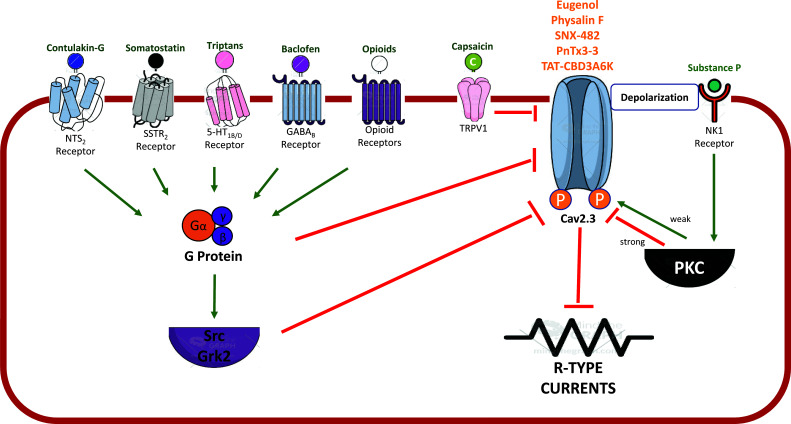
Direct or indirect inhibition of Cav2.3 by analgesic/antinociceptive drugs in cell culture. **Abbreviations**: NTS2R = Neurotensin 2 receptor; SSTR2 = Anti-somatostatin receptor type 2; HT1B/1D = 5-hidroxitriptamin receptors; GABA_B_R = gamma-aminobutyric acid B receptor; TRPV1 = transient receptor potential cation channel subfamily V member 1; NK1 = Neurokinin 1; GRK2 = G protein-coupled receptor kinase 2; PKC = Protein kinase C; Src = Proto-oncogene tyrosine-protein kinase Src; P = Phosphorilation. Figure created in the Mind the Graph platform.

Several drugs with analgesic/antinociceptive effects have been reported to inhibit depolarization evoked R-type currents directly or indirectly in cultured cells, both in primary cultures of neurons with native Cav2.3 expression (especially for the assessment of direct inhibition) or in immortalized cells heterologous expressing Cav2.3 without or with other receptors (specially to assess indirect inhibition) (Table **3** and Fig. **2**). The plant phenol Eugenol is a unique drug that is able to produce analgesia in human patients (usually in dentistry) [47] and was reported to directly block R-type currents, independent of TRPV1 interactions (Table **3**) [48]. Moreover, certain peptides purified from spider venoms (such as the toxins SNX-482 and PnTx3-3) [49], endogenous molecules (such as L-cysteine) [50], peptides (such as TAT-CBD3A6K) [51] as well as the plant steroid Phylasin F [52] were reported to produce antinociception in rodent models of pain (Table **5**) and to directly block R-type currents, with toxins typically being the more selective blockers (Table **3**). Of note, clinical studies indicate that the anticonvulsant drugs lamotrigine and topiramate may reduce neuropathic pain after spinal cord injury and prevent migraine headache attacks, respectively [53, 54]. Although both drugs are able to directly block R-type currents [55, 56], they also block several other calcium and sodium currents [57], making it difficult to determine the role of Cav2.3 on its analgesic effects.

Several drugs, such as opioids, capsaicin, zolmitriptan, baclophen somatostati, and contulakin-G, presented analgesic effects in patients with pain and can indirectly inhibit R-type currents, usually through the activation of G-protein related receptors, G-protein subunits, and protein kinase pathways [58-60] (Table **3** and Fig. **2**). Opioids are classical analgesic drugs considered the gold standard for many types of pain, and they can indirectly inhibit R-type currents [61] along with their known potent action on Cav2.2. Baclophen is clinically indicated for the treatment of spasticity and can inhibit R-type currents *via* second messenger activation [62]. The activation of the receptor for the pronociceptive neuropeptide substance (NK1) produced a dual effect: weak activation stimulated Cav2.3, whereas strong activation inhibited these channels [63]. Clinically, antagonism of NK1 receptors effectively diminishes post-operative nausea and vomiting while increasing analgesic tolerance in laparoscopic gynecological procedures [64]. Capsaicin, which is used as a topical analgesic to treat neuropathic pain in certain patients, can block R-type currents in HEK293 cells expressing Cav2.3 and TRPV1 receptors, however, the underlying mechanism is not clear [65]. Triptans, which are the gold standard to treat migraine [66], indirectly inhibit R-type currents *via* 5-HT1B receptors [67]. Somatostatin inhibits R-type currents in trigeminal rat neurons through G-protein pathways [68]. In patients with cancer pain, intrathecal somatostatin showed an analgesic effect [69]. The toxin contulakin-G can indirectly block R-type currents in DRG rat neurons [70] and showed an analgesic effect in patients with central neuropathic pain [71]. Although these various compounds and drugs do produce inhibition of Cav2.3 calcium channels, they often have multiple other targets, and thus, it remains unclear to what extent their analgesic actions can be attributed to the inhibition of Cav2.3 channels.

### 3.3 Cav2.3 Modulation of Nociceptive Transmission

Another category for the involvement of Cav2.3 in pain is the effect of Cav2.3 inhibition on nociceptive transmission. All but one out of eight findings in this regard were obtained using the selective blocker SNX-482 (Table **4**). Four studies applied SNX-482 to DRG neurons, causing excitability of intact type-A (highly myelinated and touch-pressure-vibration sensitive) neurons and reducing depolarization-activated Ca^++^ transients and R-currents in small (unmyelinated C nociceptive) neurons obtained from naïve rats (Table **4**) [72, 73]. Nerve injury reduced the SNX-482 effect on calcium transients, but not its effect on whole-cell currents [74]. There is a subpopulation of DRG neurons that communicate with their immediate neighbors *via* trans glia, (neuron-glial cell-neuron) transmission (a.k.a. sandwich synapses) [75]. This transmission was unaffected by SNX-482 (Table **4**). On the other hand, SNX-482 application in spinal cord slices reduced the frequency of spontaneous postsynaptic currents, including excitatory currents, indicating a pre-synaptic site of action at glutamatergic synapses [38]. Moreover, PKC activation increased sPSC frequency, an effect reduced by SNX-482 and by Cav2.3 global gene deletion [76]. Spinal SNX-482 and PnTx3-3 application in anesthetized rats exerted dose-dependent inhibition of noxious C-fiber- and Aδ-fiber-mediated neuronal responses in conditions of neuropathy, but not in sham-operated animals [77, 78]. Moreover, responses to innocuous mechanical and thermal stimuli were more sensitive to SNX-482 in nerve injury than in sham-operated animals. Finally, intrathecal SNX-482 inhibited the depolarization of rat colonic afferent neurons, especially in animals with colon inflammation induced by TNBS [36]. In summary, there is clear evidence that Cav2.3 channels are important mediators of the function of neurons within the primary afferent pain pathway, and this fits with the idea that these channels are important for nociceptive signaling.

### 3.4 Cav2.3 Modulators on Non-clinical (Animal) Models of Pain

Mice and rats were the only experiment animals used in non-clinical models of pain, including models of physiological nociceptive pain (*e.g*., using tail-flick, hot plate- and von Frey in naïve animals), inflammatory nociceptive pain (formalin, TNBS, CFA) and traumatic or drug-induced neuropathic pain (partial sciatic nerve or spinal nerve ligation traumatic models, streptozotocin, stavudine, paclitaxel and capsaicin) (Table **5**).

Regarding physiological nociceptive pain models in naïve mice, mechanical pain threshold (withdrawal threshold to von Frey filaments) was unchanged by Cav2.3 global knockout as well as by spinal (i.t.) knockdown (ASO) or block (SNX-482 and PnTx3-3) (Table **5**). On the other hand, knocking down Cav2.3 in the DRG of naïve mice by intra-ganglion injection of a specific AAV-shRNA reduced such thresholds (*e.g*., caused hyperalgesia) as well as reduced Cav2.3 protein in DRG.

Three studies investigated the role of Cav2.3 in models of inflammatory somatic pain induced by formalin. Cav2.3 KO or block with i.t. SNX-482 reduced spontaneous nocifensive responses in the 2^nd^ phase of formalin-induced acute nociception in both mice and rats, an effect related to reduced SP release (NK1 internalization) and neuronal activation (c-fos) in superficial laminae of the spinal cord dorsal horn. The second phase of the formalin test is considered a model of central sensitization, demonstrating that Cav2.3 may participate in the induction of central sensitization [3]. Regarding inflammatory visceral pain, one study indicated that i.t. SNX-482 reduced mechanical hyperalgesia induced by intracolonic TNBS [42]. Finally, there are conflicting findings concerning the effect of Cav2.3 global gene deletion in the model of somatic-visceral nociception induced by intraperitoneal acetic acid. Whereas initial results demonstrated no alteration of nociception compared to wild-type mice [41], a recent study indicated increased nociception [61], suggesting that Cav2.3 activation usually produces a nociceptive effect in somatic and visceral inflammatory pain.

The role of Cav2.3 was also addressed in neuropathic pain models. Systemic, but especially intrathecal, Cav2.3 blockers (SNX-482, PnTx3-3, TAT-CBD3A6K, physalin F) were able to reduce mechanical hyperalgesia in rodent models of neuropathy related to diabetes (streptozotocin), to nerve trauma (sciatic or spinal nerve ligation) and to HIV or cancer treatment (stavudine and paclitaxel). On the other hand, Cav2.3 global or local (spinal cord and DRG) knockout were not capable of altering mechanical hyperalgesia in a model of traumatic neuropathic pain in mice, an event that may be related to a compensatory increase in N- and L-type currents [79]. Additionally, local Cav2.3 (spinal cord and DRG) knockout reversed the anti-hyperalgesic effect of neurotensin, but not morphine, in the traumatic model of neuropathy. It is known that members of the Cav2 family are indirectly regulated by GPCRs, including opioid receptors. There is some evidence that morphine can have a better analgesic effect when combined with a blocker of Cav2.3 [23, 80]. However, Cav2.3 block and knockdown (ASO) are capable to inhibit, with greater efficacy in female mice, the secondary hyperalgesia induced by capsaicin, a model of central sensitization [81].

The tools used in the study of Cav2.3 in pain pathways are quite limited. There is just one selective blocker of Cav2.3 channels SNX-482, however, it also acts on other ion channels at higher doses [82-84]. Global knockout for Cav2.3 is a good tool, but this type of ablation causes a compensatory effect in other channels [79]. An alternative method is the use of local knockouts for Cav2.3 using antisense, CRISPR/Cas9 or siRNA systems, although the use of these systems requires some knowledge in the development of stable sequences, with degradation resistance and a good delivered system [85, 86]. Due to these, new more selective Cav2.3 blockers and more effective tools for the downregulation of this channel are needed to better probe the role of this channel in pain.

Methodological quality and transparent reporting improve the standards by which animal research is conducted [90]. Considering animals’ studies and pain models to measure the participation of Cav2.3, we used some criteria of methodological quality to analyse the animal studies [30]. Of the 13 animal studies, 92% indicated the experimental sample size (N) used, 62% of these studies reported that they blinded the experiments, and just 31% of studies indicated that they randomized the experiments. One parameter that is considered a good indication of the quality of the study is the description of animal welfare. However, many details regarding housing conditions and timing outcome assessment are often unreported, and the current report based on the quality of animal studies is poor [91]. Just 46% of studies described the temperature of the cages of the animals, and 54% did not mention this parameter. Besides, 69% of the studies described a conflict of interest by the authors. When we analysed the type of animals used, 46% of these studies used mice and 54% used rats. Regarding the sexes used in these studies, 23% of the studies did not mention the sex of the animals used; among those that described the sex, 31% used male, 8% used female, and 38% used both sexes. Just 23% of these studies reported all parameters (Table **6**).

These animals’ studies are important to understand the participation of Cav2.3 in different types of pain and the mechanisms involved in their development and maintenance. However, most of these studies demonstrated deficiencies in the description of study design quality which is a limitation of these studies.

### 3.5 Cav2.3 Polymorphism Association with Clinical Painful Conditions

We found 3 studies involving humans. Two of them showed that there is an association between a polymorphism in *CACNA1E* gene and phenotypes of postoperative pain. The rs3845446 single-nucleotide polymorphism (SNP) in the CACANA1E gene is an intronic tag SNP in the linkage disequilibrium block from intron 46 to exon 47, a region that contains a stop codon. Patients with the minor G allele of the rs3845446 single-nucleotide polymorphism in *CACNA1E* gene required less opioid for pain control, in a study with 355 Japanese patients who underwent painful orthognathic cosmetic surgery [92]. On the other hand, after gastrointestinal surgery, the same polymorphism in *CACNA1E* led to higher opioid requirements and increased pain scores [93]. These suggest that a single polymorphism in the *CACNA1E* gene can influence the analgesic effects of opioids, with opposite associations depending on the source of pain (somatic *vs*. visceral).

A third clinical study with healthy individuals and migraine patients demonstrated a single polymorphism rs35737760 in CACNA1E is more prevalent in hemiplegic and brain stem aura migraine. This variant causes a change from aspartate to glutamate at position 859 of Cav2.3 protein, and this polymorphism can modulate the function of R-type calcium [94].

The polymorphisms seen in these clinical studies are ambiguous because evidence shows that the increase in the activity of Cav2.3 cause nociception, and in another way, the decrease/block in Cav2.3 activity causes nociception. It appears that Cav2.3 can modulate pain in different ways depending on the type of pain and underlying mechanisms that are involved.

## CONCLUSION

The role of Cav2.2 channels in pain pathways has been extensively studied, but the role of Cav2.3 channels has remained under-explored. Our analysis of the available literature suggests that there is dysregulation of Cav2.3 channels in the pain pathway of certain preclinical pain models in rodents. There is clear evidence that inhibition of Cav2.3 channels activity affects the electrophysiological properties of neurons in the afferent pain pathway in a manner that is largely consistent with a pronociceptive role of these channels, and this is to some extent backed up by studies involving deletion of Cav2.3 channels. There is also a good correlation between the analgesic effects of a number of different compounds and drug molecules and their ability to either directly r indirectly inhibit Cav2.3 channel activity. However, except for SNX-482, there is no selective inhibitor of Cav2.3 channels, and hence it remains unclear whether the observed analgesic effects of direct or indirect Cav2.3 channel inhibitors are indeed mediated by an action on these channels or other targets. Finally, there is emerging evidence of genetic mutations in *CACNA1E* that may be consistent with the role of Cav2.3 in pain signalling in humans. However, unlike in the case of seizure disorders [95], there is no gain of function mutations identified in patients with chronic pain conditions. In this context, it is interesting to note that gain of function mutations in *CACNA1E* that lead to epilepsy do not apparently cause persistent pain, but this can perhaps be explained by the existence of different splice variants of Cav2.3 with different susceptibility to functional alterations that may be differentially expressed in the brain versus the peripheral nervous system. Overall, we conclude that still more work is needed that will allow a clear-cut identification of the role of Cav2.3 channels in the transmission and processing of peripheral pain signals. The development of small organic selective inhibitors of Cav2.3 would be of tremendous utility in addressing this issue.

## Figures and Tables

**Table 1:** Final search strategy used.

**Search Strategy for PubMed**	**Descriptors**
For Cav2.3 intervention:	(“Calcium Channels, R-Type”[Mesh] OR “cav2.3” OR “CACNA1E” OR “α1e” OR “r-type channels” OR “R-type calcium channel” OR “class E calcium channel” OR “α1e” OR “alpha1e” OR “R-type Ca^2+^ channels” OR “R-type channel” OR “R-channel” OR “R-type calcium channels” OR “CaV2.3” OR “CaV2.3 calcium channels” OR “Cav2.3 calcium channels” OR “Cav2.3” OR “calcium channel alpha 1e” OR “R-type calcium channel”)
For pain outcome:	(“pain” [MeSH Terms] OR analgesic OR analgesia OR nociception OR nociceptive OR antinociceptive OR antinociception OR antihyperalgesic OR hyperalgesia OR hypersensitivity OR hyposensitivity OR allodynia OR “neuropathic pain” OR “neuropathic” OR “Von-Frey filament” OR “vonFrey” OR “von frey”)
**Search Strategy for Scopus**	**Descriptors**
For Cav2.3 intervention:	cav2.3 OR voltage-gated calcium channel r-type OR voltage-dependent calcium channel r-type OR voltage-gated calcium channel Cav2.3 OR voltage-dependent calcium channel Cav2.3 OR VGCC r-type OR calcium channel r-type OR VGCC Cav2.3 OR voltage-sensitive calcium channel r-type OR VSCC r-type OR CACNA1E OR α1e OR r-type channels OR R-type calcium channel OR class E calcium channel OR R-type channel OR R-type calcium channels OR CaV2.3 calcium channels OR Cav2.3 calcium channels OR Cav2.3 R-type Ca^2+^ channel OR calcium channel alpha 1e OR R-type calcium channel
For pain outcome:	pain OR analgesic OR analgesia OR nociception OR nociceptive OR antinociceptive OR antinociception OR antihyperalgesic OR hyperalgesia OR hypersensitivity OR hyposensitivity OR allodynia

**Table 2:** Cav2.3 expression in pain pathways and its alteration by painful conditions.

**Localization**	**Study**	**Critical Findings**	**Painful Conditions**
Sensory Ganglia	(Castro *et al.* 2017) [36]	Co-expression of Cav2.3 (mRNA and protein) with GABA_B_R and Cav2.2 in human and mouse colonic DRG neurons	SNX-482 or Vc1.1 inhibited afferent depolarization in naïve and chronic visceral hypersensitivity mice (see table **4**)
	(Fang *et al.* 2007; 2010) [24, 25]	Co-expression of Cav2.3 (mRNA) with TrkA and TRPV1 in small rat trigeminal and DRG neurons	Not studied
	(Gandla *et al.* 2017) [37]	Cav2.3 protein was expressed in most of the neurons (PGP+) and a few satellite cells (GFAP+) of mouse DRG. Co-expression of Cav2.3 (protein) was seen with IB4, SP and NF200 in mouse DRG neuron	Expression of Cav2.3 (protein) was reduced by the pronociceptive microRNA-34c-5p in the DRGs isolated from tumor-bearing mice (see table **5**)
	(Martin *et al.* 2021) [38]	Co-expression of Cav2.3 (mRNA) with NTSR2 in mouse DRG neurons	Cav2.3 gene deletion did not alter mechanical hyperalgesia, but reversed the anti-hyperalgesic effect of somatostatin without altering morphine effects in the PSNL model (see table **5**)
	(Murakami *et al.* 2001) [39]	Cav2.3 mRNA was detected in mouse DRG	SNX-482 caused antinociception in rodent models of pain [40] (see table **5**)
	(Saegusa *et al.* 2000) [41]	Cav2.3 reporting gene (Gal) was expressed in DRG neurons, co-expressed with SP or IB4	Cav2.3 knockout did not alter nociception induced by mechanical or heat stimuli in naïve animals and by acetic-acid, but reduced both phases of formalin-induced spontaneous nociception (see table **5**)
	(Qian *et al.* 2013) [42]	Expression of Cav2.3 (mRNA and protein) and R-type currents were increased in the DRGs isolated from rats with TNBS-induced inflammatory visceral hypersensitivity	SNX-482 intrathecal injection attenuates visceral pain in TNBS-induced inflammatory visceral hypersensitivity (see table **5**)
Spinal cord	(Martin *et al.* 2021) [38]	Cav2.3 protein was especially detected in the presynaptic fraction of the spinal cord	Expression or distribution of Cav2.3 protein was unchanged in the spinal dorsal horn of mice with partial sciatic nerve ligation
	(Murakami *et al.* 2001) [39]	Cav2.3 protein was expressed in mouse dorsal horn and column	SNX-482 caused antinociception in rodent models of pain (Murakami *et al.* 2004) (see table **4**)
	(Saegusa *et al.* 2000) [41]	Cav2.3 gene expression was seen in mouse dorsal horn (laminae I-III) along the entire cord, co-expressed in IB4+ and IB4- terminals	Cav2.3 global gene deletion did not alter nociception induced by mechanical or heat stimuli in naïve animals and by acetic-acid, but reduced both phases of formalin-induced spontaneous nociception (see table **5**)
	(Westenbroek *et al.* 1998) [43]	Cav2.3 protein was mainly expressed in the soma of neurons in the dorsal horn of rats	Not studied
	(Yang *et al.* 2004) [44]	Cav2.3 mRNA was detected in the dorsal horn of the spinal cord of naïve rats	Expression of Cav2.3 mRNA was the most strongly up-regulated ion channel in rat dorsal horn spinal cord after axotomy
Supraespinal structures	(Saegusa *et al.* 2000) [41]	Cav2.3 reporting gene expressed in the periaqueductal gray, but not in nucleus raphe magnus of mice	Not studied
	(Yokoyama *et al.* 1995) [45]	Cav2.3 protein exhibited the most prominent staining in thalamus, hypothalamus, and amygdala of rats	Not studied
Enteric nervous system	(Needham *et al.* 2010) [46]	Cav2.3 protein expressed in intrinsic primary afferent neurons of guinea pigs	Not studied

**Abbreviations:** mRNA = messenger RNA; GABA_B_R = gamma-aminobutyric acid B receptor; DRG = dorsal root ganglion; TrkA = Tropomyosin receptor kinase A; TRPV1 = transient receptor potential cation channel subfamily V member 1; PGP = Protein gene product; GFAP = Glial fibrillary acidic protein; IB4 = Isolectin B4; SP = Substance P; NF200 = Neurofilament 200; NTSR2 = Neurotensin receptor 2; TNBS = 2,4,6-Trinitrobenzenesulfonic acid.

**Table 3:** Direct or indirect inhibition of depolarization evoked R-type currents in cultured cells by analgesic/antinociceptive drugs.

**Cell Type**	**Study**	**Critical Findings**	**Implication to Pain**
**Direct Interactions**			
HEK293 cells expressing Cav2.3 without or with TRPV1	(Chung *et al.* 2008) [60] (Mohammadreza *et al.* 2022) [61]	The plant phenol eugenol inhibits and blocks R-type currents, an effect unaltered by TRPV1 expression L-cysteine increased the amplitudes of recombinant Cav2.3 currents	Topical eugenol is used as an anesthetic/analgesic in dentistry L-cysteine lowered visceral pain in WT mice, and the effect was abolished in KO mice (see table **5**)
DRG and TG Rat and mouse neurons	(Fang *et al.* 2007; Murakami *et al.* 2004) [24, 40]	Toxin SNX-482 selectively inhibited R-type current with variable efficacy among different neurons	SNX-482 caused antinociception in rodent models of pain (see table **5**)
Cerebellar granule rat neurons	(Leão *et al.* 2000) [62]	The toxin PnTx3-3 unselectively inhibited R-type > N-type currents	PnTx3-3 caused antinociception in rodent models of pain (see table **5**)
DRG rat neurons	(Piekarz *et al.* 2012) [63]	The synthetic peptide TAT-CBD3A6K inhibited R-type and T-type currents in small-diameter neurons	TAT-CBD3A6K caused antinociception in rodent models of pain (see table **5**)
	(Shan *et al.* 2019) [64]	The plant steroid Physalin F inhibited N- > R-type currents	Phylasin caused antinociception in rodent models of pain (see table **5**)
**Indirect Interactions**			
Xenopus oocytes co-expressing Cav2.3 and μ--opioid receptor (rat)	(Bourinet *et al.* 1996) [65]	A selective agonist of μ-opioid receptor did not alter Cav2.3- currents	Opioids are gold-standard analgesic drugs to treat several types of pain
Xenopus oocytes co-expressing Cav2.3 and μ-opioid receptor (murine)	(Ottolia *et al.* 1998) [66]	A selective agonist of μ-opioid receptors inhibited Cav2.3 current through the voltage- and G protein-dependent pathway	Opioids are gold-standard analgesic drugs to treat several types of pain
HEK293 cells co-expressing human Cav2.3, μ, δ-, or κ-opioid receptors	(Berecki *et al.* 2016) [67]	Selective agonists of μ-, δ-, and κ-opioid receptors inhibited Cav2.3 currents through voltage-independent as well as G protein βγ subunit and GRK2 kinase-dependent pathways	Opioids are gold-standard analgesic drugs to treat several types of pain
HEK293 cells co-expressing human Cav2.3 and GABA_B_ receptor	Berecki *et al.* 2014) [68]	Baclofen or alpha-conotoxin Vc1.1 inhibited Cav2.3 channels through voltage-independent pathways by acting on GABA_B_ receptors; the modulation involves G protein- and Src kinase-dependent pathways	Baclofen was analgesic in patients with severe spasticity [54] Vc1.1 caused antinociception in a rodent pain model (see table **5**)
HEK293 cells co-expressing human Cav2.3 channels and NK1 receptor	(Meza *et al.* 2007) [69]	Weak NK1 receptor activation stimulated Cav2.3 through a PKC-dependent pathway, whereas strong activation elicited Cav2.3 inhibition	Neurokinin-1 receptor antagonism reduced post-operative pain [55]
HEK293 cells expressing Cav2.3 and TRPV1	(Chung *et al.* 2008) [60]	The plant alkylamide capsaicin inhibited R-type current through a TRPV1-dependent pathway	Topical capsaicin is analgesic in patients with neuropathic pain [56]
HEK293 cells expressing Cav2.3	(Morikawa *et al.* 2006) [70]	Zolmitriptan indirectly inhibited P/Q-type > R-type currents through 5-HT1B/1D receptor- and Gi/o-dependent pathways	Triptans are gold-standard analgesic drugs to treat migraine [57]
Trigeminal rat neurons	(Mehrke *et al.* 1997) [71]	Somatostatin inhibited Cav2.3-gated currents through a G protein-dependent pathway	Intrathecal and epidural somatostatin produced analgesia in humans with cancer or postoperative pain [58]
DRG rat neurons	(Martin *et al.* 2021) [38]	The toxin contulakin-G indirectly inhibited R-type > N-type currents through NTSR2	Contulakin-G was analgesic in patients with spinal cord injury [59]

**Abbreviations:** GRK2 = G protein-coupled receptor kinase 2; PKC = Protein kinase C; NK1 = Neurokinin 1; 5-HT1B/1D = 5-hidroxitriptamin receptors; Src = Proto-oncogene tyrosine-protein kinase Src.

**Table 4:** Cav2.3 modulation of nociceptive transmission.

**Cell or Tissue (Specie)**	**Study**	**Conditions**	**Main Findings**
DRG A-type neurons/cell membrane potential (Rat)	(Lirk 2008) [75]	Naïve	SNX-482 caused neuronal excitability
DRG neurons/depolarization-activated neurotransmitter release (Chicken)	(Rozanski *et al.* 2013) [72]	Naïve	SNX-482 did not alter transmitter release at sandwich synapses
DRG neurons/depolarization-activated Ca^++^ transients (Rat)	(Fuchs *et al.* 2007) [76]	Sham-operated and Spinal nerve ligated	SNX-482 decreased calcium transients in small neurons of naïve rats, with reduced sensitivity in neuropathic rats
DRG neurons/depolarization-activated R-type current (Rat)	(McCallum 2011) [77]	Sham-operated and Spinal nerve ligated	SNX-482 decreased current in small neurons of naïve rats, without altered sensitivity in neuropathic rats
Spinal cord/spontaneous excitatory postsynaptic currents (sEPSCs) (Mice)	(Martin *et al.* 2021) [38]	Naïve	SNX-482 reduced sEPSCs, especially their frequency
Spinal cord/spontaneous postsynaptic currents (sPSCs) (Rat)	(Yang *et al.* 2013) [73]	Naïve	SNX-482 reduced sEPSC frequency in dorsal horn neurons. PKC activation increased sEPSC frequency, an effect reduced by SNX-482 and by Cav2.3 global gene deletion
Spinal cord/spontaneous excitatory postsynaptic currents (sEPSCs) (Rat)	(Shan *et al.*, 2019) [64]	Naïve	Physalin F reduced the frequency of sEPSCs
Dorsal horn neuronal electrophysiological measurements (Rat)	(Matthews *et al.* 2007) [78]	Sham-operated and Spinal nerve ligated	Spinal SNX-482 inhibited noxious C-fibre- and Aδ-fiber-mediated neuronal responses in conditions of neuropathy, but not in sham-operated animals
	(Dalmolin *et al.* 2017) [74]	Sham-operated and Spinal nerve ligated	Spinal PnTx3-3 inhibited noxious C-fiber- and Aδ-fiber-mediated neuronal responses in conditions of neuropathy, but not in sham-operated animals
Colonic afferents depolarization electrophysiological measurements (Mice)	(Castro *et al.* 2017) [36]	Naïve and TNBS-treated rat	SNX-482 or Vc1.1 inhibited afferents depolarization

**Abbreviations:** sPSCs = spontaneous postsynaptic currents; sPSCs = spontaneous excitatory postsynaptic currents.

**Table 5:** Cav2.3 modulators on non-clinical (animal) models of pain.

**Specie/Models**	**Study**	**Critical Behavior Finding**	**Supporting *Ex vivo* Findings**
Mice/von Frey, hot-plate, tail- and paw-flick-, formalin- and acetic acid	(Saegusa *et al.* 2000) [41]	Cav2.3 global gene deletion did not alter nociception induced by mechanical or heat stimuli in naïve animals and by acetic acid, but reduced both phases of formalin-induced spontaneous nociception	None
Mice/Von Frey	(Gandla *et al.* 2017) [37]	Intraganglion injection of Cav2.3 knocking down (AAVs-shRNA Cav2.3) caused hyperalgesia	Cav2.3 AAVs-shRNA reduced the expression of Cav2.3 protein in DRG
Mice/Tail-flick	(Yokoyama *et al.* 2004) [80]	Cav2.3 global gene deletion in mice resulted in greater analgesia to heat stimuli by morphine or by swim-stress (and resistance to morphine tolerance) than in controls	None
Mice/Formalin	(Murakami *et al.* 2004) [40]	Intrathecal SNX-482 inhibited the second phase, but increased the first phase of nociception	None
Rat/Formalin	(Terashima *et al.* 2013) [87]	Intrathecal SNX-482 inhibited both phases of nociception	Neurokinin 1 receptor internalization and c-Fos expression in the ipsilateral dorsal horn
Rat/TNBS	(Qian *et al.* 2013) [42]	Intrathecal SNX-482 reduced the abdominal withdrawal reflex in response to colorectal distention	None
Mice/Acetic acid	(Mohammadreza *et al.* 2022) [61]	Cav2.3 global gene deletion increased acetic acid-induced nociception; an effect reduced by systemic L-cysteine	L-cysteine increases R-type currents
Mice/Partial sciatic nerve ligation (PSNL)	(Yang *et al.* 2009) [88]	Cav2.3 global gene deletion in mice did not alter the development of mechanical hyperalgesia	PSNL reduced the R-type current in DRG neurons and Cav2.3 global gene deletion increased N- and L-type currents
	(Martin *et al.* 2021) [38]	Cav2.3 local (DRG and spinal cord) gene deletion did not alter the development of mechanical hyperalgesia, but reversed the anti-hyperalgesic effect of neurotensin without altering morphine’s effect	None
Rat and mice/PSNL and streptozotocin	(Dalmolin *et al.* 2011) [89]	Intrathecal PnTx3-3 reduced mechanical hyperalgesia in neuropathic rodents, but not in naïve animals	None
Rat/Stavudine	(Piekarz *et al.* 2012) [63]	Intraperitoneal TAT-CBD3A6K produced antinociception in a rodent model of pain	None
Rat/Paclitaxel and spinal nerve ligation	(Shan *et al.* 2019) [64]	Intrathecal *phylasin* F produced antinociception in rodent models of neuropathy	None
Mice/ Capsaicin	(Ferreira *et al.* 2021) [81]	Intrathecal SNX-482 or Cav2.3 ASO caused antinociception in the secondary hyperalgesia trigged by capsaicin in a sex-dimorphic way and SNX-482 or Cav2.3 ASO did not alter the nociception induced by mechanical or heat stimuli in naïve animals	Cav2.3 ASO reduced Cav2.3 mRNA expression in the spinal cord

**Abbreviations:** AAVs-shRNA = Adeno-Associated Virus short hairpin RNA; CFA = Complete Freund’s Adjuvant; PSNL = Partial sciatic nerve ligation; TNBS = 2,4,6-trinitrobenzenesulfonic acid.

**Table 6:** Quality analyses of the animal studies.

**Study**	**N**	**Blind**	**Random**	**Temper.**	**Conflict**	**Specie**	**Sex**
Dalmolin 2011	OK	OK	-	OK	OK	Rats	M and F
Ferreira 2021	OK	OK	OK	OK	OK	Mice	M and F
Gandla 2017	OK	-	-	-	-	Mice	-
Martin 2021	OK	OK	OK	OK	OK	Rats	M and F
Mohammadreza 2022	OK	OK	-	-	OK	Mice	Male
Murakami 2004	-	-	-	OK	-	Mice	-
Piekarz 2012	OK	-	OK	OK	OK	Rats	Female
Qian 2013	OK	OK	-	-	OK	Rats	Male
Saegusa 2000	OK	OK	-	-	-	Mice	M and F
Shan 2019	OK	OK	OK	OK	OK	Rats	Male
Terashima 2013	OK	OK	-	-	OK	Rats	Male
Yang 2009	OK	-	-	-	OK	Mice	-
Yokoyama 2004	OK	-	-	-	-	Mice	M and F

**Abbreviations:** N = number per experimental group, OK = the information described in the paper, - = no information found in the paper.

## LIST OF ABBREVIATIONS

HVAs: High-voltage-activated Channels
LVA: Low-voltage Activated
NSAIDs: Nonsteroidal Anti-inflammatory Agents
SNP: Single-nucleotide Polymorphism
SNRIs: Serotonin and Norepinephrine Reuptake Inhibitors
VGCCs: Voltage-gated Calcium Channels

## STANDARDS OF REPORTING

PRISMA guidelines and methodology were followed.

## SUPPLEMENTARY MATERIAL

PRISMA checklist is available as supplementary material on the publisher’s website along with the published article.
